# Periodontal disease does not increase the risk of subsequent psoriasis

**DOI:** 10.1038/s41598-023-32907-8

**Published:** 2023-04-12

**Authors:** Yoo Sang Baek, Eun-Jung Kwak, Young Chan Kim, Ko Eun Kim, Hae Jun Song, Jiehyun Jeon

**Affiliations:** 1 Department of Dermatology, Guro Hospital, Korea University College of Medicine, 148 Gurodong-ro, Guro-gu, Seoul, 08308 Republic of Korea; 2 National Dental Care Center for Person with Special Needs, Seoul National University Dental Hospital, Seoul, Republic of Korea

**Keywords:** Diseases, Medical research, Risk factors

## Abstract

Previous studies suggested that chronic periodontitis may be a risk factor for psoriasis. However, no study has confirmed this relationship for all stages of periodontal disease (gingivitis and periodontitis). This nationwide population-based retrospective cohort study aimed to investigate whether periodontal disease is an independent risk factor for the development of subsequent psoriasis. Patients aged ≥ 20 years who underwent both medical and oral checkups from the National Health Screening Program between 2002 and 2007 were selected from a customized database provided by the National Health Insurance Service (NHIS). Then, patients with periodontal disease (*n* = 3,682,468) and without periodontal disease (control, *n* = 3,637,128) according to oral examination results were identified. We tracked each patient for subsequent psoriasis diagnosis until the end of 2018 using NHIS database. The incidence rates of psoriasis per 1000 person-years were 0.36 and 0.34 in the periodontal disease group and control groups, respectively. After adjusting for potential cofactors, no significant increase in risk (adjusted hazard ratio, 0.994; 95% confidence interval, 0.974–1.015) was observed. Similar results were observed when analyzing the risk of psoriasis in patients who required scaling or periodontal surgery. In conclusion, periodontal disease is not an independent risk factor of psoriasis.

## Introduction

Psoriasis is an immune-mediated chronic inflammatory skin disease associated with a substantial physical and psychological burden^1^. Its global prevalence ranges from 0.2 to 4.8%^2^. Psoriasis is known to be associated with multiple comorbidities, such as psoriatic arthritis, cardiovascular disease, metabolic syndrome (obesity, diabetes, hypertension, and dyslipidemia), and psychiatric disorders^1^.

Periodontal disease is a group of inflammatory conditions that affect the gingiva, bone, and periodontal ligaments that provide structural support to the teeth^3,4^. Periodontal disease usually starts with gingivitis, which is a mild, reversible, and localized inflammation of the gum that is initiated by a microbial biofilm (dental plaque)^3,4^. The condition progresses to periodontitis when the inflammation extends deeper and causes the loss of supporting tissue, which creates periodontal pockets^3,4^. The prevalence of periodontal disease varies based on the disease definition and study population^3–5^. A surveillance study in the United States showed that 47.2% of adults aged ≥ 30 years suffer from some form of periodontitis^6^.

Currently, investigators believe that periodontal diseases (gingivitis and periodontitis) share common etiopathogenesis, and gingivitis almost always precedes periodontitis^3,4^. In addition, the absence of gingivitis is a good indicator of long-term periodontal health in an individual^3,7^. Furthermore, periodontal disease is not limited to the oral space but is an inflammatory condition associated with systemic diseases such as diabetes and cardiovascular diseases^3,4^. Numerous studies have reported association between periodontal disease (mainly, chronic periodontitis) and psoriasis^8–11^. Shared genetic factors, common pathophysiology, and risk factors have been suggested to explain this association^11,12^. For instance, increase level of interleukin (IL)-17 has been implicated in common pathophysiological link between two entities^12^. Elevated levels of IL-17 in psoriatic patients systemically inhibits osteoblast and induces osteoclast activities, resulting in low bone formation and bone loss^13,14^. This process may theoretically contribute to the development of periodontitis^13^. On the other hand, *Porphyromonas gingivalis* in chronic periodontitis can activate the Th17 pathway, and the levels of locally produced IL-17 increased in periodontitis patients^13,15,16^. Therefore, increased level of IL-17 in psoriasis and periodontitis may generate a vicious cycle^12^.

Moreover, recent studies have shown that periodontal disease may be an independent risk factor for psoriasis^17–19^. However, this suggestion can be confounded by common risk factors (such as smoking, diabetes, and obesity) of both disease. A recent study in Korea investigated the risk of psoriasis in patients with periodontal disease who had been diagnosed with chronic periodontitis and visited dental clinics^17^. Since patients with early periodontal disease as well as chronic periodontitis rarely seek professional dental care^3,4^, this recent study may have selected patients with advanced stages who sought clinical care. Therefore, there is a lack of evidence for relationship between psoriasis and general periodontal disease (including gingivitis and periodontitis), regardless of dental visit status. We hypothesized that overall periodontal disease is an independent risk factor for subsequent psoriasis. To capture early or asymptomatic periodontal disease patients, we used oral examination results from the National Health Screening Program (NHSP) for the detection of periodontal disease.

## Materials and methods

### Data source and study population

We conducted a nationwide population-based retrospective cohort study using a customized database provided by the National Health Insurance Service (NHIS). The NHIS is a single non-profit insurer in Korea that covers insurance for almost the entire Korean population. It also offers standardized biennial medical and oral checkups under the NHSP. The NHIS manages a computerized database with healthcare-related information about individuals as well as their personal information. The NHIS provides a customized database to researchers with de-identified information regarding the study population upon request.

In order to acquire as many individuals as possible, we requested a customized database of all patients aged ≥ 20 years who underwent both medical and oral checkups on the same day (the index date) from 2002 and 2007. In the customized database, we collected medical and oral checkup results, personal information (sex, birth year, and death year), as well as medical claims data of the participants from the index date to the end of 2018. The medical diagnoses in the claims data were defined using the International Classification of Disease, 10th Revision (ICD-10) codes, which is an internationally-recognized classification system that provides standardized codes for each disease^20^. We excluded the participants who had missing relevant data, died during the study period, or were diagnosed with psoriasis before their index date.

### Data collection and interpretation

All participants completed standardized medical and oral checkups, answered questionnaires, and underwent clinical and laboratory assessments. The self-reported questionnaire collected data on smoking habits, alcohol consumption, and personal or familial medical history. Height and weight were measured and body mass index (BMI) was calculated by dividing weight (kg) by height squared (m^2^). In accordance with the national manual for oral health examination^21^, trained dentists performed the clinical oral examinations using necessary dental examination tools, such as a dental mirror and probe. The results were recorded using a standardized evaluation form. This form consisted of multiple check boxes, such as the presence of dental caries, missing teeth, and dental abrasion. The dentist confirmed the diagnosis of periodontal disease when severe calculus or the presence of periodontal pockets were observed during clinical oral examination^21^. This professional opinion was then used as a proxy for the participants' periodontal status^22^. Furthermore, they recorded the observed clinical features like gingival hyperplasia, dental plaque, and periodontal pockets that suggested the presence of periodontal disease. Multiple answers were allowed for the observed clinical features. In the last section “Final opinion”, the dentist checked whether the participant required scaling or periodontal surgery. Periodontal surgery is generally suggested when the periodontal disease is too severe to be corrected by non-surgical approach such as scaling^3,21^.

A diagnosis of psoriasis was defined as at least two documented visits to a dermatologist with a diagnosis of psoriasis (L40) from the index date until the end of the study. We excluded the diagnosis of other clinical variants of psoriasis (L40.1, generalized pustular psoriasis; L40.2, acrodermatitis continua; L40.3, pustulosis palmaris et plantaris; and L40.4, guttate psoriasis). Patients were considered to have an underlying disease (diabetes or hypertension) if they had been diagnosed with the corresponding ICD-10 code during the one-year period prior to the index date.

### Statistical analysis

Descriptive statistics for continuous variables, such as age, are presented as means with standard deviation, and their distribution is assessed for normality using the Shapiro–Wilk test and Kolmogorov–Smirnov test. Categorical variables are presented as frequencies with percentages in parentheses. The baseline characteristics of the study groups were compared using Student’s *t*-test for continuous variables and Pearson’s chi-squared test for categorical variables. The incidence of psoriasis was calculated by dividing the total number of incident cases by the entire follow-up duration (person-years). The Cox proportional hazards model was used to calculate hazard ratios (HR) for developing new psoriasis in participants with periodontal disease. Analyses were adjusted for the following cofactors; age at index date, sex, smoking status (never or ever), diabetes, obesity (BMI ≥ 30 or < 30)^23^, heavy alcohol consumption (≥ 3 times per week), and hypertension. In model 1, adjustment for age and sex was performed. Due to the frequent mention of smoking, diabetes, and obesity as common risk factors for both periodontal disease and psoriasis^3,4,24–26^, we conducted additional adjustment for these factors in model 2. The remaining potential confounding factors (hypertension and heavy alcohol consumption)^27,28^ were adjusted for in model 3. We performed subgroup analyses based on the presence of clinical features suggesting periodontal disease, such as gingival hyperplasia, dental plaque, and periodontal pockets. Furthermore, in our additional statistical analysis, we calculated the incidence rate and hazard ratio of subsequent psoriasis among participants who required scaling or periodontal surgery. The statistical significance level was set at *α* = 0.05. All statistical analyses were performed using SAS^®^ (SAS Institute, Cary, NC, USA).

### Ethics

The study protocol was approved by the Institutional Review Board (IRB) of the Korea University Guro Hospital (2020GR0594), which waived the requirement for informed consent. The National Health Information Data Request Review Committee approved the study protocol and use of NHIS data for research (NHIS-2021-1-321). This study was conducted in accordance with the principles of the Declaration of Helsinki.

## Results

### Study population and baseline characteristics

**Figure 1:**
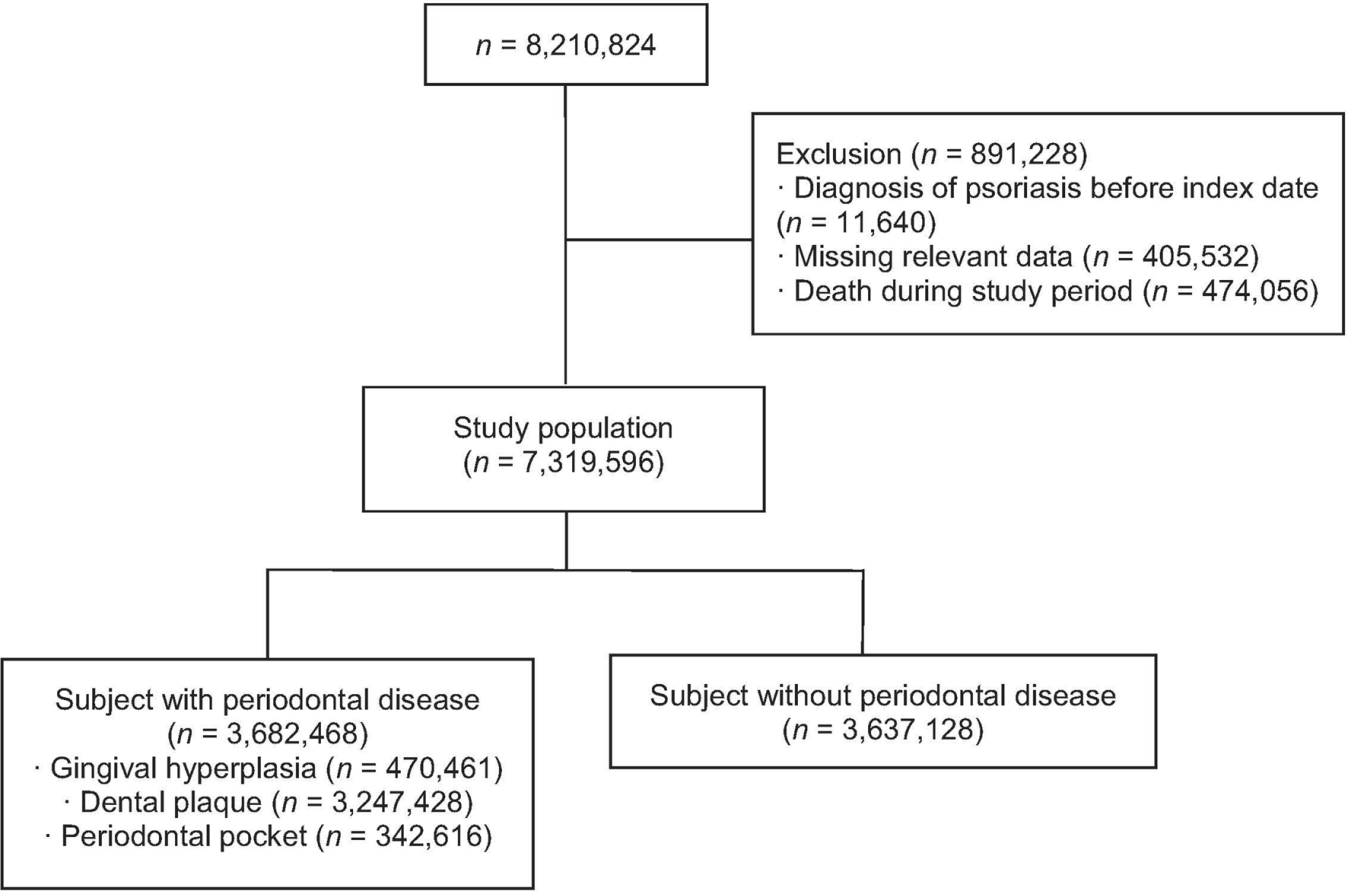
A flow chart of the study cohort selection.

From 2002 to 2007, a total of 8,210,824 individuals underwent both medical and oral checkups on the same day (the index date). After exclusion, a total of 7,319,596 individuals were included in this study. An oral examination revealed that 3,682,468 participants (50.3%) had periodontal disease. Regarding the observed clinical features suggesting periodontal disease, 470,461 participants had gingival hyperplasia, 3,247,428 had dental plaque, and 342,616 had periodontal pockets (Fig. 1). The baseline characteristics of participants with and without periodontal disease are shown in Table 1. The age distribution was found to be normal according to the Shapiro–Wilk and Kolmogorov–Smirnov tests (*p* > 0.05). Patients with periodontal disease were more likely to be of higher age, male, smokers, diabetic, obese, heavy drinkers, and hypertensive compared to those without periodontal disease (*p* < 0.0001). According to the final opinion of the examining dentist, 3,619,014 participants required scaling, and 187,450 participants required periodontal surgery.

**Table 1:** Comparison of baseline characteristics between the study groups with and without periodontal disease.

	Without periodontal disease (n = 3,637,128)	With periodontal disease (n = 3,682,468)	*p*-value
Mean age ± SD, years	40.22 ± 13.00	41.83 ± 12.42	< 0.0001
Sex, male, n (%)	1,989,752 (54.71)	2,353,664 (63.92)	< 0.0001
Smoking, ever smoker, n (%)	1,245,860 (34.25)	1,616,190 (43.89)	< 0.0001
Diabetes, n (%)	155,069 (4.26)	179,172 (4.87)	< 0.0001
Obesity, n (%)	89,388 (2.46)	112,866 (3.06)	< 0.0001
Heavy alcohol consumption, yes, n(%)	249,742 (6.87)	360,572 (9.79)	< 0.0001
Hypertension, n (%)	310,404 (8.53)	347,109 (9.43)	< 0.0001

*SD* standard deviation.

### Incidence rate and risk of psoriasis among participants with periodontal disease

The average duration of the follow-up period is 14.2 years. During the follow-up period, the incidence rate of subsequent psoriasis in the participants with periodontal disease was 0.36 cases per 1,000 person-years; it was 0.34 cases per 1000 person-years in participants without periodontal disease. The unadjusted HR showed a significantly higher risk of psoriasis in patients with periodontal disease (HR, 1.051; 95% confidence interval [CI] 1.029–1.073). However, after adjusting for age and sex, there was no significant increase in the risk (adjusted HR, 1.010; 95% CI 0.990–1.032). After adjusting for other potential cofactors (smoking, diabetes, obesity, heavy alcohol consumption, and hypertension), the adjusted HR decreased further (adjusted HR, 0.994; 95% CI 0.974–1.015) (Table 2). The incidence rate of psoriasis among individuals with observed clinical features that suggesting periodontal disease (gingival hyperplasia, dental plaque, or periodontal pockets) was 0.34–0.36 per 1000 person-years. After adjusting for age and sex, there was no significant increase in the risk of subsequent psoriasis in patients with clinical features suggestive of periodontal disease (Supplementary Table 1).

**Table 2:** The incidence rate and risk of incidence of subsequent psoriasis by study group.

	Events	Follow-up duration, person-years	Incidence rate	Unadjusted HR (95% CI)	Adjusted HR in model 1 (95% CI)	Adjusted HR in model 2 (95% CI)	Adjusted HR in model 3 (95% CI)
Presence of periodontal disease							
Without periodontal disease (n = 3,637,128)	17,637	51,890,444.62	0.34	1	1	1	1
With periodontal disease (n = 3,682,468)	18,718	52,402,411.31	0.36	1.051 (1.029–1.073)	1.010 (0.990–1.032)	0.994 (0.974–1.015)	0.994 (0.974–1.015)
Requiring scaling							
No need for scaling (n = 3,700,582)	17,892	52,782,320.8	0.34	1	1	1	1
Need for scaling (n = 3,619,014)	18,463	51,510,535.14	0.36	1.057 (1.036–1.079)	1.017 (0.996–1.038)	1.001 (0.980–1.022)	1.001 (0.980–1.022)
Requiring periodontal surgery							
No need for periodontal surgery (n = 7,132,146)	35,417	101,618,023.98	0.35	1	1	1	1
Need for periodontal surgery (n = 187,450)	938	2,674,831.95	0.35	1.006 (0.943–1.074)	0.962 (0.902–1.027)	0.948 (0.889–1.012)	0.949 (0.889–1.013)

*HR* hazard ratio, *CI* confidence interval.

Model 1: adjusted for age and sex.

Model 2: adjusted for model 1 plus smoking, diabetes, and obesity.

Model 3: adjusted for model 2 plus heavy alcohol consumption, and hypertension.

### Incidence rate and risk of psoriasis among participants requiring scaling or periodontal surgery

The incidence rate of subsequent psoriasis in participants who required scaling was 0.35 cases per 1,000 person-years, compared with 0.34 in those who did not require scaling. The unadjusted HR showed a significantly higher risk of psoriasis in participants who required scaling (HR, 1.057; 95% CI 1.036–1.079). However, after adjusting for age and sex, there was no significant increase in the risk (adjusted HR, 1.017; 95% CI 0.996–1.038). After adjusting for other cofactors, the adjusted HR further decreased (Table 2).

The incidence rate of subsequent psoriasis in participants who needed periodontal surgery was 0.35 cases per 1000 person-years, compared to 0.35 in those who did not require periodontal surgery. There was no significant increase in the risk of subsequent psoriasis in participants who required periodontal surgery before and after adjustment for cofactors (Table 2).

## Discussion

Periodontal disease only occurs in susceptible hosts when there is an imbalance between the host’s immune system and oral dysbiosis, which refers to the loss of the diversity and balance of the oral microbiota^3,4,13,29^. Early periodontal disease has no or mild symptoms, and patients usually visit the dental clinics in the advanced stages of periodontal disease^3,4^. Non-surgical removal of dental plaque (scaling and root planning) can be initially performed, but surgical procedures such as open flap surgery may be required for advanced periodontal disease^3,4^. In contrast to previous studies suggesting periodontal disease, particularly advanced forms, may be an independent risk factor for psoriasis^17–19^, our study found no evidence of an increased risk, both in early and advanced periodontal disease.

After the first report by Yamada et al*.*^30^, the association between psoriasis and periodontal disease (especially chronic periodontitis) was studied in multiple case-control^12,31–38^ and cohort studies^17–19,39,40^. Although there are studies showing a non-significant association after adjusting for cofactors^32,36,40^, recent meta-analyses and systematic reviews have confirmed a positive relationship between the two disease entities^8–11^. Thus, individuals with psoriasis were more likely to have periodontitis than those without psoriasis^12,31,33–35,38,39^. Moreover, compared with controls, patients with psoriasis showed significantly worse periodontal clinical parameters such as plaque index^31,32^, bleeding on probing^12,31,32,34^, probing depth^31–33^, clinical attachment level^31,32^, or community periodontal index^12^. However, studies showed great heterogeneity in the diagnosis of periodontal disease and evaluation of periodontal status. In our current study, the professional opinion by dentists during oral examination was used a proxy for periodontal status. Unfortunately, due to limited data in the oral examination results, periodontal clinical parameters were not available.

The relationship between psoriasis and periodontal disease has been explained by relevant genetic factors, shared pathophysiology, and common risk factors^11,12^. In particular, genetic polymorphisms for interleukin (IL)-1, IL-6, and tumor necrosis factor-α have been linked to both psoriasis and periodontal disease, respectively^41–45^. Additionally, both diseases are considered to be inflammatory conditions that are caused by an aberrant activation of the innate and adaptive immune systems due to possible triggering factors like oral dysbiosis in the case of periodontitis^13,31^. Finally, smoking, diabetes, and obesity have been demonstrated as common risk factors for both diseases^3,4,24,25^.

Some cohort studies have shown that periodontal disease (mainly chronic periodontitis) is an independent risk factor for subsequent psoriasis^17–19^. Using the Taiwanese administrative health database, Keller et al.^18^ reported that the risk of subsequent psoriasis in patients with chronic periodontitis was greater than that in the control group during a five-year period. However, they did not adjust for the effects of cigarette smoking, which is an important confounding factor. Nakib et al.^19^ used prospective cohort data to show that individuals with a history of periodontal bone loss had an increased risk of psoriasis. The identification of both psoriasis and periodontitis in their study was self-reported without validation from a clinician. Furthermore, Han et al.^17^ used the Korean NHIS database to show that chronic periodontitis is an independent risk factor for psoriasis and smoking plays a synergistic role in this regard. In their study, they identified chronic periodontitis patients using medical claims data^17^. Therefore, only patients with official dental visits were studied. In their study, approximately 10.9% of the study population was identified as having chronic periodontitis^17^, which corresponds to the reported prevalence (10–15%) of advanced forms in epidemiological data^3^. Therefore, we could speculate that their study population only included selected patients with advanced stages who sought dental care.

Contrary to our initial hypothesis, this study failed to show that periodontal disease was an independent risk factor for psoriasis. The unadjusted HR was significant. However, this significance was lost after adjusting for age and sex. Similar results were observed when analyzing the risk of psoriasis in participants who required scaling (indicative of periodontal disease) or periodontal surgery (indicative of an advanced status of periodontitis). Based on the results of our study and previous relevant studies, we believe that periodontal disease is a comorbidity of psoriasis but that general periodontal disease itself is not an independent risk factor for subsequent psoriasis.

There are several possible explanations for this discrepancy between the results of the previous cohort studies and our study. First, only advanced forms of periodontal disease (severe chronic periodontitis) may serve as risk factors for psoriasis, not the early forms (gingivitis or mild-to-moderate chronic periodontitis). However, in our study, participants who needed periodontal surgery, which is an indicator of an advanced stage did not have a significantly high risk of psoriasis, suggesting a limited explanation. Another possibility is that participants became aware of their poor periodontal health after oral checkups and may have received appropriate dental care. This may have attenuated the effects of periodontal disease on psoriasis. Notably, Keller et al.^18^ showed that periodontitis patients treated with gingivectomy or periodontal flap operation had a lower risk of developing psoriasis than that in periodontitis patients who did not receive such treatment. Furthermore, studies have shown that local treatment of periodontal disease can ameliorate systemic inflammatory markers and improve surrogate markers of certain systemic diseases (i.e., diabetes)^29^. Additionally, previous cohort studies with positive results could be victims of a surveillance bias, with chronic periodontitis patients being more likely to be diagnosed with psoriasis based on their increased exposure to dental/medical communities. Finally, most relevant studies are observational epidemiological studies prone to confounding and reverse causation. In fact, a recent Mendelian randomization study, which is known to be free of confounding or reverse causation, failed to show any effect of periodontitis on psoriasis, or vice versay^46^. Therefore further detailed prospective studies and experimental studies are required to clarify whether periodontal disease (severe or not) is an independent risk factor for psoriasis.

The limitations of the current study include the use of the ICD-10-based administrative NHIS database without a direct review of individual medical records and the lack of data on certain relevant information such as periodontal clinical parameters, medication status, and psoriasis severity. This could limit the integrity of the periodontal disease or psoriasis diagnosis. Additionally, our study is susceptible to selection bias as it only includes patients who underwent both medical and oral checkups, and thus those who did not participate in these checkups were not included in the study. Nonetheless, the use of a large general population (7.3 million) and the long follow-up period (an average of 14.2 years) are strengths of our study. In addition, multiple possible cofactors were considered. Therefore, the result of this study are valuable for researchers investigating the relationship between poor oral hygiene and psoriasis.

## Conclusion

While previous studies have suggested that periodontal disease, specifically chronic periodontitis, may be an independent risk factor for psoriasis, our study found no evidence to support this claim. None of the oral examination results indicating periodontal disease were statistically associated with a significant increase in the risk of subsequent psoriasis. Consequently, it appears unnecessary to conduct routine clinical examinations of periodontal disease patients for possible psoriasis diagnosis. Further studies are needed to clarify the relationship between psoriasis and periodontal disease.

## Supplementary Information

Supplementary Table 1.

## Data Availability

The data that support the findings of this study are available from National Health Insurance Sharing Service (NHISS) but restrictions apply to the availability of these data, which were used under license for the current study, and so are not publicly available. Data are however available from the authors upon reasonable request and with permission of NHISS. Contact the corresponding author for request or any further information.
