# Supplementary material for: Periodontal disease does not increase the risk of subsequent psoriasis
Source: Sci Rep. 2023 Apr 12;13:5942. doi: 10.1038/s41598-023-32907-8 (PMC10097622; doi:10.1038/s41598-023-32907-8)
Supplement: Supplementary file 1 — Supplementary Table 1. [file 41598_2023_32907_MOESM1_ESM.docx]

**Supplementary Table 1.** The incidence rate and risk of incidence of subsequent psoriasis by study group (without periodontal disease and with clinical features that suggested periodontal disease (gingival hyperplasia, dental plaque or periodontal pockets))

|  | Events | Follow-up duration, person-years | Incidence rate | Unadjusted HR (95% CI) | Adjusted HR in model 1 (95% CI) | Adjusted HR in model 2 (95% CI) | Adjusted HR in model 3 (95% CI) |
| --- | --- | --- | --- | --- | --- | --- | --- |
| Without periodontal disease  (n=3,637,128) | 17,637 | 51,890,444.62 | 0.34 | 1 | 1 | 1 | 1 |
| With gingival hyperplasia (n=147,290) | 755 | 2,193,551.95 | 0.34 | 1.013  (0.942–1.072) | 0.985  (0.916–1.059) | 0.975  (0.907–1.049) | 0.975  (0.906–1.049) |
| With dental plaque (n=2,800,431) | 14,282 | 39,744,546.89 | 0.36 | 1.057  (1.034–1.081) | 1.016  (0.994–1.039) | 1.000  (0.978–1.022) | 1.000  (0.978–1.023) |
| With periodontal pocket (n=147,868) | 726 | 2,145,059.53 | 0.34 | 0.996  (0.924–1.072) | 0.951  (0.883–1.025) | 0.937  (0.870–1.010) | 0.938  (0.871–1.010) |

*HR* hazard ratio, *CI* confidence interval

Model 1: adjusted for age and sex

Model 2: adjusted for model 1 plus smoking, diabetes, and obesity

Model 3: adjusted for model 2 plus heavy alcohol consumption, and hypertension
